# Hedgehog Signaling Promotes the Proliferation and Subsequent Hair Cell Formation of Progenitor Cells in the Neonatal Mouse Cochlea

**DOI:** 10.3389/fnmol.2017.00426

**Published:** 2017-12-21

**Authors:** Yan Chen, Xiaoling Lu, Luo Guo, Wenli Ni, Yanping Zhang, Liping Zhao, Lingjie Wu, Shan Sun, Shasha Zhang, Mingliang Tang, Wenyan Li, Renjie Chai, Huawei Li

**Affiliations:** ^1^ENT Institute and Otorhinolaryngology Department, Affiliated Eye and ENT Hospital, State Key Laboratory of Medical Neurobiology, Fudan University, Shanghai, China; ^2^Key Laboratory of Hearing Medicine of National Health and Family Planning Commission (NHFPC), Shanghai, China; ^3^Key Laboratory for Developmental Genes and Human Disease, Ministry of Education, Institute of Life Sciences, Southeast University, Nanjing, China; ^4^Jiangsu Province High-Tech Key Laboratory for Bio-Medical Research, Southeast University, Nanjing, China; ^5^Co-innovation Center of Neuroregeneration, Nantong University, Nantong, China; ^6^Institutes of Biomedical Sciences, Fudan University, Shanghai, China; ^7^The Institutes of Brain Science and the Collaborative Innovation Center for Brain Science, Fudan University, Shanghai, China; ^8^Shanghai Engineering Research Centre of Cochlear Implant, Shanghai, China

**Keywords:** inner ear, Hedgehog signaling, hair cell regeneration, Lgr5, proliferation, differentiation

## Abstract

Hair cell (HC) loss is the major cause of permanent sensorineural hearing loss in mammals. Unlike lower vertebrates, mammalian cochlear HCs cannot regenerate spontaneously after damage, although the vestibular system does maintain limited HC regeneration capacity. Thus HC regeneration from the damaged sensory epithelium has been one of the main areas of research in the field of hearing restoration. Hedgehog signaling plays important roles during the embryonic development of the inner ear, and it is involved in progenitor cell proliferation and differentiation as well as the cell fate decision. In this study, we show that recombinant Sonic Hedgehog (Shh) protein effectively promotes sphere formation, proliferation, and differentiation of Lgr5+ progenitor cells isolated from the neonatal mouse cochlea. To further explore this, we determined the effect of Hedgehog signaling on cell proliferation and HC regeneration in cultured cochlear explant from transgenic R26-SmoM2 mice that constitutively activate Hedgehog signaling in the supporting cells of the cochlea. Without neomycin treatment, up-regulation of Hedgehog signaling did not significantly promote cell proliferation or new HC formation. However, after injury to the sensory epithelium by neomycin treatment, the over-activation of Hedgehog signaling led to significant supporting cell proliferation and HC regeneration in the cochlear epithelium explants. RNA sequencing and real-time PCR were used to compare the transcripts of the cochleae from control mice and R26-SmoM2 mice, and multiple genes involved in the proliferation and differentiation processes were identified. This study has important implications for the treatment of sensorineural hearing loss by manipulating the Hedgehog signaling pathway.

## Introduction

Hair cell (HC) loss is the major cause of sensorineural hearing loss, and unlike lower vertebrates, mammalian cochlear HCs cannot regenerate spontaneously after damage, although the vestibular system does maintain limited HC regeneration capacity. Thus, promoting HC regeneration in the sensory epithelium is a promising strategy to restore hearing after HC loss caused by ototoxic drugs, noise exposure and genetic defects.

The Hedgehog pathway is a highly conserved signaling pathway that plays critical roles during the early developmental stages of the inner ear, including the proliferation of progenitor cells and their subsequent cell fate determination and differentiation. Most vertebrate species have three Hedgehog genes (Sonic Hedgehog (*Shh*), Indian Hedgehog (*Ihh*) and Desert Hedgehog (*Dhh*)). The transmembrane protein Patched (Ptc) serves as a ligand of Hedgehog protein, and in the presence of Hedgehog Ptc activity is repressed resulting in the de-repression of another transmembrane protein, Smoothened (Smo), and thus initiating the signaling cascade. This results in the activation of glioma-associated oncogene *Gli* transcription factors (in vertebrates) and the expression of Hedgehog target genes (Gorojankina, 2016).

Multiple studies have demonstrated that Hedgehog signaling plays important and complicated roles during the development of the inner ear, and inactivation of Hedgehog signaling leads to the mis-regulation of proliferation and differentiation in the mammalian cochlea during development (Riccomagno et al., 2002; Driver et al., 2008; Liu et al., 2010; Brown and Epstein, 2011; Bok et al., 2013; Son et al., 2015). Our previous study showed that Shh protein promotes the proliferation and HC differentiation of mouse embryonic inner ear prosensory cells (Zhao et al., 2006). However the role of Hedgehog signaling in regulating HC regeneration in the postnatal mouse cochlea has not been well investigated, and the mechanism behind the effects of Hedgehog signaling in regulating HC regeneration need to be further investigated.

It has been reported that Wnt-responsive Lgr5+ supporting cells are HC progenitor cells in the mouse inner ear (Chai et al., 2012; Shi et al., 2012; Li et al., 2016; Waqas et al., 2016a,b; Cheng et al., 2017; Zhang et al., 2017). Lgr5+ cells isolated by flow cytometry from neonatal Lgr5^EGFP–CreERT2^ mice can proliferate to form clonal colonies and can mitotically regenerate new HCs (Chai et al., 2012; Waqas et al., 2016a; Cheng et al., 2017; Zhang et al., 2017). Promoting the proliferation and differentiation of Lgr5+ progenitor cells thus appears to be a promising strategy to mitotically regenerate HCs. Our previous study showed that Wnt activation and Notch inhibition stimulate the proliferation of Lgr5+ cells and promote the mitotic regeneration of HCs (Chai et al., 2012; Wang et al., 2015; Ni et al., 2016a,b; Wu et al., 2016).

Previous studies report that the Hedgehog pathway is important to the formation of proliferating Müller glia-derived progenitor cells during chicken retinal regeneration (Todd and Fischer, 2015). Activation of Hedgehog signaling via constitutively active Smo results in both normal and neoplastic cerebellar growth through up-regulation of N*-myc* (Kenney et al., 2004; Hatton et al., 2006). Considering the important role of Hedgehog signaling in inner ear development, in this article we investigated the effects and the mechanism of Hedgehog signaling on the proliferation and differentiation of postnatal cochlear Lgr5+ progenitor cells. We found that the activation of Hedgehog signaling promoted the proliferation of Lgr5+ progenitor cells and subsequent HC differentiation. In cultured cochlear explants from the Sox2^CreERT2/+^ R26SmoM2 mice, in which Hedgehog signaling is up-regulated in Sox2+ supporting cells by supplying 4-OH tamoxifen in the culture medium, we found that Hedgehog signaling activation significantly increased the proliferation of supporting cells and the mitotic regeneration of HCs throughout the whole length of the cochlea after HC loss induced by neomycin exposure. Lastly, the mechanism behind the increased supporting cell proliferation and HC regeneration induced by the up-regulation of Hedgehog signaling was explored. RNA sequencing and real-time PCR was used to compare the transcripts of the cochleae from control mice and R26-SmoM2 mice in Sox2+ supporting cells, and multiple genes involved in the proliferation and transdifferentiation processes were identified.

## Materials and Methods

### Mouse Models

Lgr5^EGFP–CreERT2^ (Stock 008875), Sox2^CreERT2/+^ (Stock 008875), and R26SmoM2 (Stock 005130) mice were purchased from The Jackson Laboratory. Both male and female mice were used in all experiments. This study was carried out in strict accordance with the “Guiding Directive for Humane treatment of Laboratory Animals” issued by the Chinese National Ministry of Science and Technology in September 2006. All experiments were approved by the Institutional Animal Care and Use Committee of Fudan University and the Shanghai Medical Experimental Animal Administrative Committee (Permit Number: 2009-0082). All efforts were made to minimize suffering and reduce the number of animals used.

### Sphere Culture and Differentiation Assay

We dissected the organs of Corti from postnatal day 2–3 (P2–P3) Lgr5^EGFP–CreERT2^ mice. Each organ of Corti was individually inspected, dissected from the surrounding tissue, and carefully rinsed in PBS solution. All tissues were collected in PBS at pH 7.4 on ice before they were subjected to digestion with 0.125% trypsin in PBS for 10 min at 37°C in a total volume of 140 μl. The enzymatic reaction was stopped by adding 70 μl of 20 mg/ml soybean trypsin inhibitor (Invitrogen) in DMEM/high glucose and F12 media (mixed 1:1). The cells were carefully triturated with plastic pipette tips (epTIPS Filter 20–300 μl; Eppendorf) and diluted with 2 ml DMEM/high glucose and F12 media (mixed 1:1) supplemented with N2 and B27, EGF (20 ng/ml), bFGF (10 ng/ml), and IGF-1 (50 ng/ml). Cell separation was ensured via microscopic inspection. The cell suspension was then passed through a 40 μm cell strainer directly into plastic Petri dishes (Greiner). The dissociated cells were sorted on a MoFlo^®^SX FACS cytometer (Beckman Coulter) using the channels for EGFP, and positive and negative fractions were collected. The purity of the sorted cells was assessed by re-sorting and immunostaining. A total of 2000 purified cells were plated into non-adhesive 12-well culture clusters (Costar) at a density of 2 cells/μl. Recombinant Shh (50 ng/ml, 100 ng/ml, or 200 ng/ml), cyclopamine (10 μM), SANT-1 (25 μM), or vismodegib (10 μM) was added to the culture medium. The culture medium was changed every 36 h throughout the whole culture period.

For the differentiation experiment, spheres were transferred to laminin-coated 4-well dishes, and the culture medium was replaced by DMEM/F12 media supplemented with N2 and F12 after the cells had been allowed to adhere for 24 h. Recombinant Shh (50 ng/ml, 100 ng/ml, or 200 ng/m) was added to the culture medium. The culture medium was changed every 36 h throughout the whole culture period.

For the direct differentiation assay, 2000 isolated Lgr5+ progenitor cells from P2-P3 mice were cultured in laminin-coated 4-well dishes at a density of 20 cells/μl for 7 days in serum-free medium, which is suitable for cell differentiation. Recombinant Shh (50 ng/ml, 100 ng/ml, or 200 ng/m), cyclopamine (10 μM), SANT-1 (25 μM), or vismodegib (10 μM) was added to the culture medium. The culture medium was changed every 36 h throughout the whole culture period. After 7 days of culture, the samples were fixed for immunostaining.

### Cochlear Explant Cultures

Cochleae from P2 Sox2^CreERT2/+^; R26SmoM2 transgenic mice were dissected in PBS and cultured in DMEM/F12 (Life Technologies) with 1% N2 supplement (Life Technologies), 2% B27 supplement (Life Technologies), and 50 μg/ml ampicillin (Sigma) at 37°C with 5% CO_2_. Explanted cochlear epithelium was cultured for 7 days with 4-OH tamoxifen (5 μM, Sigma-Aldrich) to induce the Cre activity and EdU (5′-ethynyl-2′-deoxyuridine, 10 μM, Life Technologies) to label proliferative cells. In the neomycin treatment group, the explanted cochlear epithelia were treated with 0.5 mM neomycin sulfate (Sigma) for the first 24 h of the culture. Cochlear explants from littermates without the SmoM2 allele served as controls.

### Immunohistochemistry

Cochlear epithelium and cells were fixed in 4% PFA (Sigma-Aldrich) for 30 min and washed with 10 mM PBS. EdU was detected with Alexa Fluorazide using the Click-iT EdU Imaging Kits (Life Technologies) according to the manufacturer’s protocol. The tissues were then blocked with 10% donkey serum in 10 mM PBS with 1% Triton X-100 (for cochlear epithelium) or 0.1% Triton X-100 (for cells) for 1 h at room temperature and incubated with primary antibody overnight at 4°C. The primary antibodies were rabbit anti-MyosinVIIa (Myo7a, 1:800 dilution; Proteus BioSciences) and goat anti-Sox2 (1:500 dilution; Santa Cruz Biotechnology). On the following day, the appropriate secondary Alexa Fluor-conjugated antibodies were incubated for 1–2 h at room temperature. Nuclei were labeled with DAPI (1:800 dilution; Sigma-Aldrich).

### RNA Sequencing and Analysis

RNA sequencing and analysis was performed as previously reported (Ni et al., 2016a). At least six cochleae from each group were pooled to isolate the total RNA using the AllPrep DNA/RNA/Protein Mini Kit (QIAGEN). Preliminary processing of raw reads was performed using Casava 1.8 (Illumina), and adapters and low-quality bases were trimmed using trimmomatic (version 0.23). Trimmed reads for each sample were aligned to the *Mus musculus* UCSC mm10 genome using the TopHat version 2.0.10 software package (Bowtie 2 version 2.2.1 software). These files were used as the input for the Cufflinks software, which is a complementary method used to generate assembled transcripts for each group, and the abundance was evaluated using read data. The fragments per kilobase per million mapped reads values were calculated for each gene to normalize the data. These assemblies were used with the Cuffdiff tools from the Cufflinks 2.2.1 package to calculate the differential expression levels and to evaluate the statistical significance of the detected alterations.

### Functional Annotation

DAVID (Database for Annotation, Visualization and Integrated Discovery) version 6.7 software^1^ was used to determine the most functional annotation of significant genes in the datasets as described previously (Huang da et al., 2009). The DAVID program calculates a modified Fisher’s exact *p*-value to demonstrate gene ontology or molecular pathway enrichment. A Benjamini-adjusted *p-*value < 0.05 was considered to be strongly enriched in the annotation category.

### Image Acquisition and Cell Counts

Fluorescent images were acquired using a Leica SP8 confocal microscope, and the contrast and brightness of the images were adjusted using Adobe Photoshop CS6. Cell counts from the confocal images were performed using ImageJ, and the numbers of Myo7a+, Sox2+ and EdU+ cells were counted in 100 μm sensory regions of the cochlea in the apical, middle and basal turns.

### Quantitative RT-PCR

At least six cochleae from each group were pooled to isolate the total RNA using the All Prep DNA/RNA/Protein Mini Kit (QIAGEN). cDNA was synthesized from 1 μg total RNA by reverse transcription using the GoScript™ Reverse Transcription System (cat. A5001, Promega). Quantitative real-time PCR (qPCR) was performed by using the GoTaq^®^ qPCR Master Mix (cat. A6001, Promega) and 7500 ReaL Time PCR system (Life Technologies). *Actb* was used as the housekeeping gene. Primer sequences are listed in Supplementary Table S1.

### Statistical Analyses

Statistical analyses were performed using GraphPad Prism software. Cell counts were analyzed with one-way ANOVA followed by a Dunnett’s multiple comparisons test when comparing more than two groups. Two-tailed, unpaired Student’s *t*-tests were used to determine statistical significance when comparing two groups. Data are shown as the means ± SE, and *p* < 0.05 was considered statistically significant.

## Results

### Hedgehog Signaling Enhances the Proliferation and Sphere-Forming Ability of Isolated Postnatal Lgr5+ Progenitors *in Vitro*

**Figure 1:**
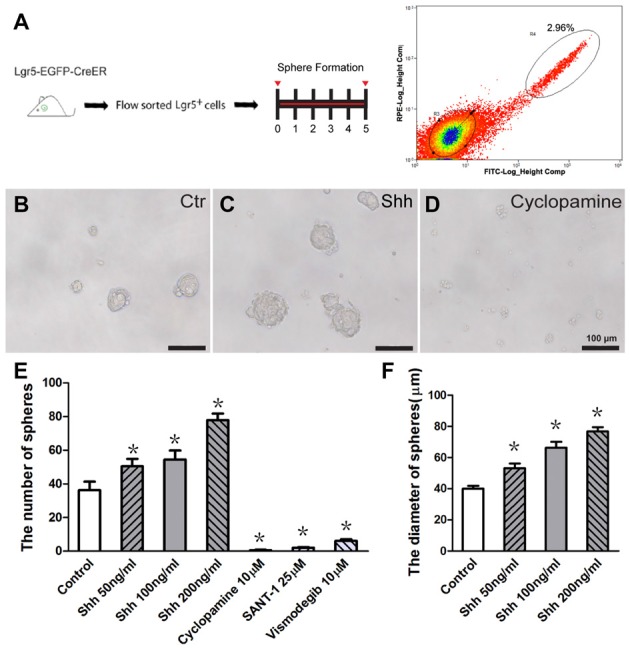
Hedgehog signaling significantly enhanced the sphere-forming ability of isolated postnatal Lgr5+ progenitor cells *in vitro*. **(A)** Diagram of the FACS and sphere-forming assay. **(B–D)** Representative images of Lgr5+ spheres in the control **(B)**, Sonic Hedgehog (Shh) protein-treated (**C**, 100 ng/ml) and cyclopamine-treated (**D**, 10 μM) groups. **(E)** The number of spheres increased significantly in the Shh protein-treated group and decreased significantly in the Hedgehog antagonist (cyclopamine, SANT-1, or vismodegib)-treated groups compared to the control group. **(F)** The average diameter of the spheres significantly increased in the Shh protein-treated group compared to the control group. Data are presented as means ± SE. *N* = 5, *p < 0.05 vs. the control group. Scale bars **(B–D)** are 100 μm.

Previous experiments have shown that Lgr5 is expressed in a subset of Sox2+ supporting cells, and Lgr5+ cells isolated by flow cytometry from the neonatal Lgr5^EGFP–CreERT2^ mouse cochlea can proliferate and form clonal colonies *in vitro* (Chai et al., 2011, 2012; Waqas et al., 2016a; Cheng et al., 2017; Zhang et al., 2017). To investigate the effect of Hedgehog signaling in regulating the proliferative ability of Lgr5+ progenitor cells, we dissociated the whole cochlear epithelium from P2–P3 Lgr5^EGFP–CreERT2^ mice into single cells and isolated the Lgr5-EGFP+ cells via flow cytometry and performed the sphere-forming assay with Hedgehog agonist and antagonist. For each experiment, 2000 sorted cells were cultured at a density of 2 cells/μl in an ultra-low attachment plate for 5 days, then the numbers and diameters of the spheres in the culture plates were determined (Figure 1A). Recombinant Shh protein was used as the Hedgehog agonist, and cyclopamine, a small molecule compound that inhibits the effects of Smo, was used as the Hedgehog antagonist. Cultured Lgr5-EGFP+ cells without Shh or cyclopamine treatment were used as controls. The isolated Lgr5+ cells generated more spheres compared with the control group when cultured with Shh (*p* < 0.05, *n* = 5), and almost no spheres were observed when cultured with cyclopamine (*p* < 0.05, *n* = 5, Figures 1B–E). The average diameter of the spheres in the Shh-treated group was significantly larger than in the control group (*p* < 0.05, *n* = 5, Figure 1F). To further verify the above results, titrated concentrations of Shh protein were used, and the number and diameter of spheres both increased as the concentration of Shh protein increased (Figures 1E,F). Moreover, another two Hedgehog signaling inhibitors, SANT-1 and vismodegib (Zarei et al., 2017), were used to further confirm the effects of Hedgehog inhibition, and the results were consistent with above data (Figure 1E). These results demonstrated that activation of Hedgehog signaling significantly promoted the cell proliferation and clone-forming ability of postnatal Lgr5+ progenitors and that inhibition of Hedgehog signaling significantly decreased the proliferative ability of Lgr5+ progenitor cells.

### Hedgehog Signaling Enhances HC Differentiation from Lgr5+ Progenitors *in Vitro*

**Figure 2:**
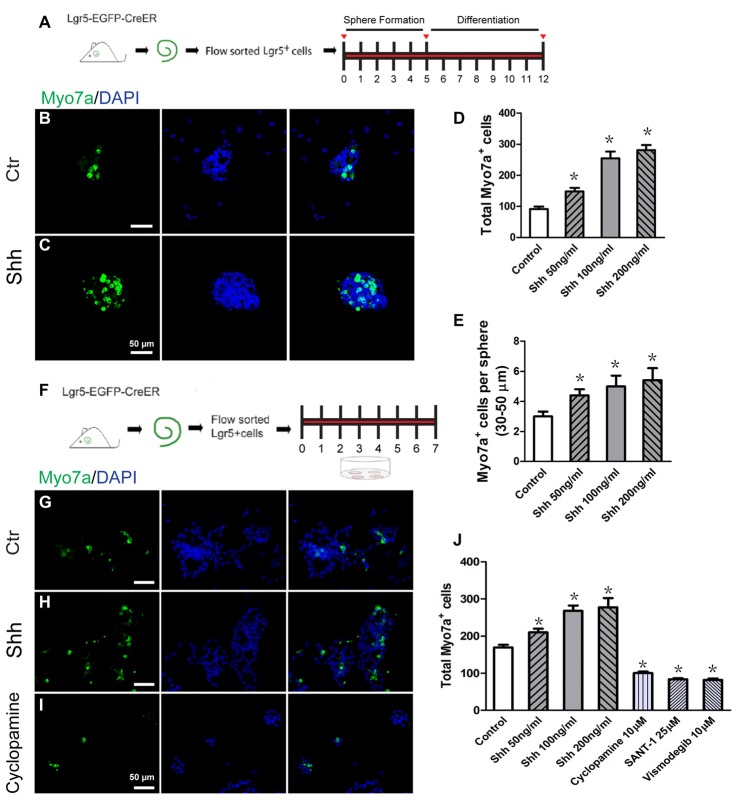
Hedgehog signaling enhanced the hair cell (HC) differentiation of inner ear progenitor cells *in vitro*. **(A)** Diagram of the differentiation assay in Lgr5+ cell spheres. After allowing spheres to form for 5 days, the spheres were collected and cultured in medium that is suitable for cell differentiation for a further 7 days. The cells were stained with antibodies against Myo7a and with DAPI after 12 days of culture. **(B)** Spheres formed by the Lgr5+ progenitor cells generated some Myo7a+ cells when cultured in differentiation medium for 7 days in the control group. **(C)** The Shh protein-treated spheres generated more Myo7a+ cells. **(D)** The total number of Myo7a+ cells per well. **(E)** The average number of Myo7a+ cells per sphere (30–50 μm) in the control and Shh protein-treated groups. **(F)** Diagram of the direct differentiation assay. A total of 2000 sorted Lgr5-EGFP+ progenitor cells were cultured on 4-well plates at a density of 20 cells/μl for 7 days in serum-free medium. **(G–I)** Representative images of Myo7a immunofluorescence after 7 days of culture in the control, Shh protein-treated, and cylcopamine-treated groups. **(J)** The number of Myo7a+ cells increased significantly in the Shh protein-treated group and decreased significantly in the Hedgehog antagonist (cyclopamine, SANT-1, or vismodegib) group when compared with the control group. Data are presented as means ± SE. *N* = 5, *p < 0.05 vs. the control group. Scale bars **(B–C,G–I)** are 50 μm.

The spheres from isolated Lgr5+ cells can differentiate into HCs as indicated by specific HC markers, and they can generate stereocilia-like structures under certain conditions (Chai et al., 2012; Waqas et al., 2016a; Cheng et al., 2017). To investigate the effect of Hedgehog signaling on HC differentiation in spheres of Lgr5+ progenitor cells, the spheres were collected and cultured in medium with Hedgehog agonist and antagonist for 7 days and then immunostained to identify the Myo7a+ HCs after 12 days of total culture (Figure 2A). The Myo7a+ HCs in each differentiated sphere as well as the total number of Myo7a+ HCs per well were calculated. Compared with the control group, the total number of Myo7a+ cells was significantly increased in the Shh-treated group (*p* < 0.05, *n* = 5; Figures 2B–D), and there were more Myo7a+ HCs in each individual sphere (30–50 μm) in the Shh-treated group than in the spheres in the control group (*p* < 0.05, *n* = 5; Figure 2E). The numbers of HCs in the spheres were increased when treated with Shh protein in a dose-dependent manner (Figures 2E,F). These results suggested that Hedgehog signaling significantly enhanced HC formation in the proliferated spheres from isolated Lgr5+ progenitor cells *in vitro*.

To further evaluate the effects of Hedgehog signaling on HC differentiation from Lgr5+ progenitor cells, we cultured 2000 isolated Lgr5+ progenitor cells in laminin-coated 4-well dishes at a density of 20 cells/μl for 7 days in serum-free medium, and these conditions are suitable for cell differentiation (Figure 2F). After 7 days of culture, the samples were fixed for immunostaining. Compared with the control group, the number of Myo7a+ cells was significantly increased in the Shh protein-treated group and significantly decreased in the Hedgehog antagonists (cyclopamine, SANT-1, or vismodegib)-treated group (*p* < 0.05, *n* = 5; Figures 2G–J). These results suggested that activation of Hedgehog signaling promoted the differentiation of postnatal Lgr5+ progenitors into HCs *in vitro* and that the differentiation of Lgr5+ progenitors to HCs is dependent on Hedgehog signaling *in vitro*.

### Hedgehog Signaling Fails to Initiate Cell Proliferation or New HC Formation in Uninjured Cochlear Sensory Epithelium

**Figure 3:**
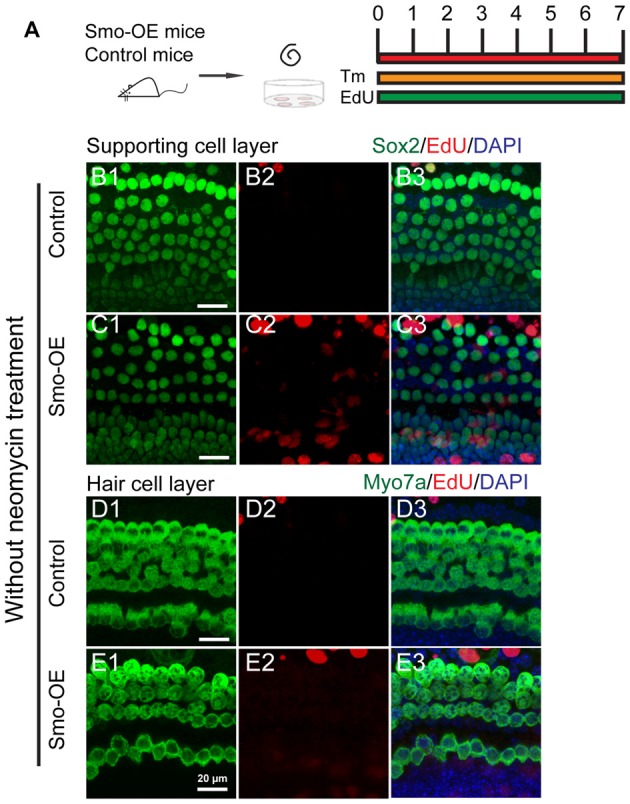
Forced activation of Hedgehog signaling did not trigger cell proliferation or new HC formation in the cochlear epithelium without neomycin treatment. **(A)** The diagram for the assay. Cochlear epithelium samples were dissected from P2 Smo-OE and control mice and then cultured in DMEM/F12 media with N2 and B27. 4-OH tamoxifen (Tm) and EdU were added throughout the culture period. At the end of 7 days of culture, the tissues were fixed and immunostained for Myo7a, Sox2 and EdU. **(B,C)** Representative immunofluorescence images of the supporting cell layer in the control and Smo-OE cochlear epithelia without neomycin treatment. No difference in the number of EdU+Sox2+ cells was seen between the Smo-OE and control groups. **(D,E)** Representative immunofluorescence images of the HC layer in the control and Smo-OE cochlear epithelia without neomycin treatment. No EdU+Myo7a+ HCs were observed in either group. Scale bars **(B–E)** are 20 μm.

To determine the effects of Hedgehog signaling on supporting cell proliferation and HC regeneration from neonatal mouse cochlear epithelium, the organ of Corti explants were cultured to evaluate *in situ* proliferation and HC generation after activation of the Hedgehog pathway. In the neonatal mouse cochleae, all the supporting cells are Sox2+, while, a small portion of the Sox2+ supporting cells, including the inner pillar cell, the third row of deiter’s cell, and the medial inner phalangeal cells are Lgr5+ (Chai et al., 2011, 2012). While, since the efficiency of Cre recombinase in Lgr5 CreER line is relatively low, we used the Sox2 CreER line in our experiments to manipulate Hedgehog pathway in supporting cells. Here we took advantage of Sox2^CreERT2/+^; R26SmoM2 transgenic mice (hereafter referred to as Smo-OE mice) in which a dominant active allele of Smo, known as SmoM2, is conditionally expressed in the Sox2+ supporting cells when Cre is activated. SmoM2 has a point mutation that prevents its negative regulation by Ptc receptor, thus leading to constitutive activation of the Hedgehog signaling pathway (Migone et al., 2016). Littermates without the SmoM2 allele were used as controls. Cochlear epithelium samples were dissected from P2 Smo-OE and control mice and then cultured in DMEM/F12 media with N2 and B27. Cre recombinase activity in Sox2+ cells was induced by 4-OH tamoxifen, and EdU was used to label proliferating cells and mitotically generated HCs (Figure 3A). After 7 days of culture, we found no significant difference in the number of EdU+Sox2+ cells between the Smo-OE and control groups (Figures 3B1–3,C1–3), and no new EdU+Myo7a+ HCs were observed in either group (Figures 3D1–3,E1–3). These results showed that activation of Hedgehog signaling could not induce cell proliferation or new HC formation in cultured intact cochlear sensory epithelium.

### Forced Hedgehog Signaling Activation Promotes Supporting Cell Proliferation and HC Regeneration after Neomycin-Induced HC Injury

**Figure 4:**
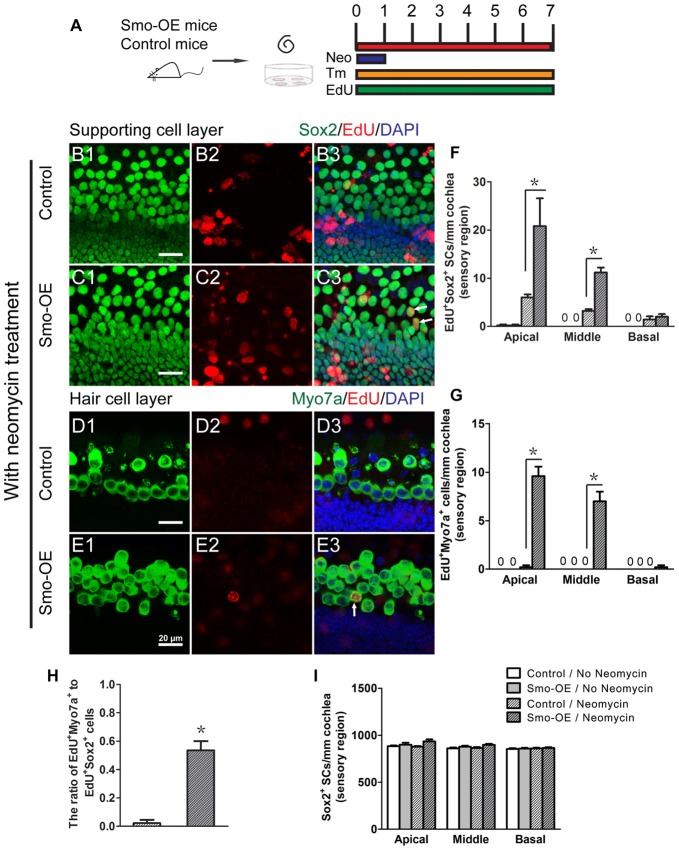
Forced activation of Hedgehog signaling promoted *in situ* supporting cell proliferation and HC regeneration after neomycin treatment. **(A)** The diagram for the assay. Cochlear epithelium samples were dissected from P2 Smo-OE and control mice and then cultured in DMEM/F12 media with N2 and B27. The explanted cochleae were treated with 0.5 mM neomycin sulfate (Neo) for 24 h. 4-OH tamoxifen (Tm) and EdU were added to the culture media throughout the entire culture period to induce the Cre activity and to label proliferating cells, respectively. **(B,C)** Representative immunofluorescence images of the supporting cell layer in the control and Smo-OE cochlear epithelia after neomycin treatment. **(D,E)** Representative immunofluorescence images of the HC layer in the control and Smo-OE cochlear epithelia after neomycin treatment. **(F)** The number of EdU+Sox2+ cells significantly increased in the Smo-OE group compared with the control group. **(G)** The number of EdU+Myo7a+ cells significantly increased in the Smo-OE group compared with the control group. **(H)** The ratio of EdU+Myo7a+ cells to EdU+Sox2+ cells in the Smo-OE and control groups. **(I)** The total number of Sox2+ supporting cells (SCs). Data are presented as means ± SE. *N* = 5, *p < 0.05 vs. the control group with neomycin treatment. Scale bars **(B–E)** are 20 μm.

During the development of the cochlea, the mosaic structure of HCs and supporting cells in the sensory epithelia is regulated through lateral inhibition by Notch signaling (Chrysostomou et al., 2012). Our previous studies demonstrated that inhibition of Notch promotes the proliferation of postnatal cochlear progenitor cells and the mitotic formation of new HCs (Li et al., 2015), thus the failure of Hedgehog activation to induce cell proliferation and HC formation in intact cochlear epithelium is likely due to lateral inhibition by Notch signaling. Therefore, neomycin treatment was used to induce HC damage and disrupt the balance of Notch signaling prior to activation of Hedgehog signaling in the supporting cells (Figure 4A). In the control group, very few EdU+Sox2+ cells were observed after neomycin-induced HC loss (Figures 4B1–3,F), and almost no newly regenerated EdU+Myo7a+ HCs were observed (Figures 4D1–3,G). Compared with the control group, the numbers of EdU+Sox2+ and EdU+Myo7a+ cells were both significantly increased in the Smo-OE group after neomycin-induced HC loss (*p* < 0.05, *n* = 5, Figures 4C1–3,E1–3,F,G), which suggested that forced activation of Hedgehog signaling in supporting cells efficiently promoted *in situ* cell proliferation and mitotic HC regeneration after neomycin-induced HC loss. Moreover, compared with the control group, the ratio of EdU+Myo7a+ cells to EdU+Sox2+ cells in the Smo-OE group also increased significantly (*p* < 0.05, *n* = 5, Figure 4H), indicating that forced Hedgehog signaling activation promotes HC differentiation in supporting cells. Most of the EdU+Myo7a+ cells were located in the pillar cell region, further supporting that the newly generated HCs derived from the Lgr5+ progenitor cells.

### Possible Mechanism behind the Effect of Hedgehog Signaling on Postnatal Lgr5+ Cells and HC Regeneration

To assess the genome-wide gene expression profiles between controls and Hedgehog activation in cochlear supporting cells, we compared the transcripts of the cochleae using RNA sequencing. The cochlear epithelia were dissected from P2 mice that had been treated with neomycin and 4-OH tamoxifen as shown in Figure 4A. A total of 6–8 cochleae were pooled for the RNA sequencing experiment.

**Figure 5:**
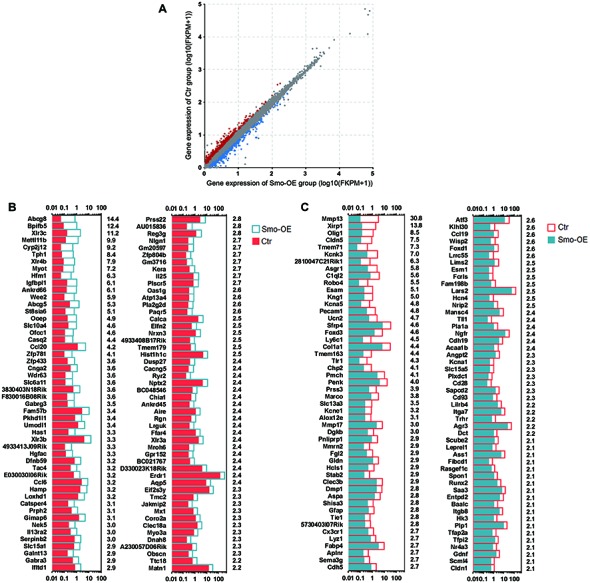
The differentially expressed genes in the Smo-OE and control cochlear epithelium. **(A)** Scatter plot for expressed genes (Log10(FKPM + 1)) in the Smo-OE and control cochlear epithelium. The dots represent differentially expressed genes between the two groups (fold change >1.5 and *p* < 0.05). The blue dots represent the highly differentially expressed genes in the Smo-OE group. The red dots represent the highly differentially expressed genes in the control group. **(B)** The top 100 highly differentially expressed genes in the Smo-OE cochlear epithelium ranked in descending order. The number on the right side of each panel represents the fold difference in expression for the Smo-OE group vs. the control group. **(C)** The top 100 highly differentially expressed genes in control cochlear epithelium ranked in descending order. The number on the right side of each panel represents the fold difference in expression for the control group vs. the Smo-OE group.

**Figure 6:**
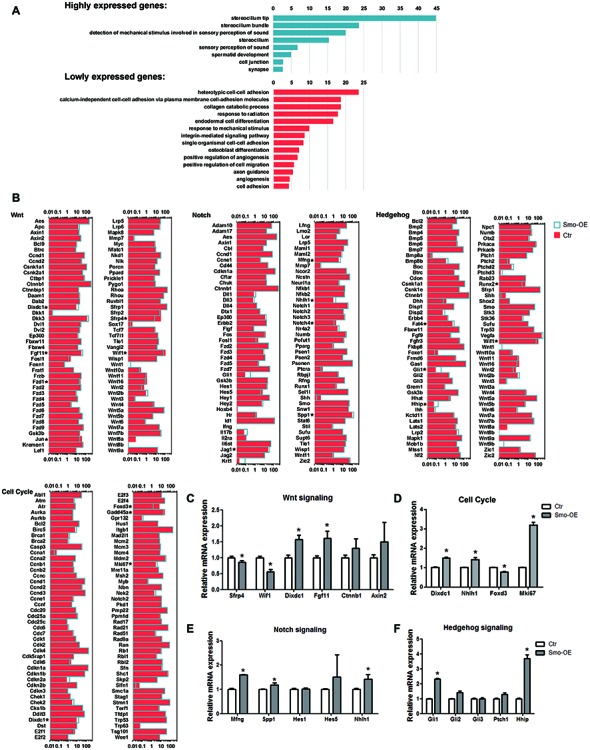
Gene ontology and signaling pathway analysis of the genes that are differentially expressed in the Smo-OE and control cochlear epithelium. **(A)** Gene ontology of the genes that are differentially expressed in the Smo-OE and control cochlear epithelium. **(B)** Signaling pathway genes in the Smo-OE and control cochlear epithelium. **(C)** q-PCR analysis of Wnt signaling pathway genes. **(D)** q-PCR analysis of cell cycle genes. **(E)** q-PCR analysis of Notch signaling pathway genes. **(F)** q-PCR analysis of Hedgehog signaling pathway genes. *p < 0.05.

A total of about 20.7–54.7 million paired reads were obtained for each sample, with at least 58% of the reads mapping correctly to the reference genome. We selected the differentially expressed genes between controls and the Smo-OE group by comparing their expression levels (fold change > 1.5, *p* < 0.05). We found 329 genes that were significantly up-regulated and 222 genes that were significantly down-regulated in the Hedgehog-activation group (Figures 5, 6A,B). Real-time PCR was performed to confirm the RNA sequencing results (Figures 6C–F), and the two results were consistent.

Among these differentially expressed genes between the control and Smo-OE groups, we found that *Gli1* and *Hhip*, two downstream targets of Hedgehog signaling, were significantly up-regulated in the Smo-OE group, further confirming the constitutive activation of Hedgehog signaling in the cochlear supporting cells of Smo-OE mice.

Compared with the control group, there were two up-regulated (*Mki67* and *Dixdc1*) and two down-regulated (*Foxd3* and *Gadd45a*) cell cycle-related genes in the Smo-OE group. *Mki67*, which encodes the nuclear protein ki67 (a marker for proliferating cells; Lin et al., 2016), was significantly up-regulated in the Hedgehog-activation group.

There were 30 up-regulated and 11 down-regulated transcription factors in the Smo-OE group. For example, *Nhlh1*, which encodes a helix-loop-helix protein, was up-regulated in the Smo-OE group, and *Foxd3*, a tumor suppressor (Schmid and Müller, 2013), was down-regulated in the Smo-OE group.

Multiple signaling pathways are involved in regulating the proliferation, differentiation, and cell fate determination of inner ear progenitor cells. Compared with controls, 12 up-regulated and 26 down-regulated signaling pathway-regulating genes were observed in the Smo-OE group, including *Wif1*, *Sfrp4*, *Dixdc1*, *Mfng*, *Nhlh1*, *Fgf11*, *Spp1*, *Ptc1* and *Gli1*. Two negative regulators of Wnt signaling, *Wif1* and *Sfrp4*, were significantly down-regulated in the Smo-OE group. *Mfng*, a negative regulator of Notch signaling (Veeraraghavalu et al., 2004; Golson et al., 2009), was up-regulated in the Smo-OE group. *Fgf11*, *Dixdc1*, *Nhlh1* and *Spp1* were also up-regulated in the Smo-OE group.

## Discussion

Hedgehog signaling plays critical roles in the self-renewal capacity of stem cells, the cell fate determination of progenitor cells, and the tonotopic organization of HCs during inner ear development (Son et al., 2015). The mouse inner ear derives from a thickened ectodermal region adjacent to the hindbrain, referred to as the otic placode, at embryonic day 8.0 (E8). At E8–E12, Shh is highly expressed in the notochord, floor plate, and cochlear vestibular ganglion (Bok et al., 2013). Shh is first expressed in all cochlear spiral ganglion neurons by E13.5, after which its expression gradually decreases from the base to the apex of the cochlea. By postnatal day 0 (P0), the expression of Shh is no longer detectable in any spiral ganglion neurons (Liu et al., 2010). It is reported that ventral otic derivatives, including the cochlear duct and cochlear vestibular ganglia, fail to develop in *Shh−/−* embryos. Each of these morphological defects is related to alterations in the expression of otic vesicle patterning genes (Riccomagno et al., 2002). Brown generated embryos in which Smo was conditionally inactivated in the otic epithelium (Smo(ecko)) and found that ventral otic identity is directly dependent on Hedgehog signaling. Ventral otic derivatives failed to form in Smo(ecko) embryos, whereas vestibular structures developed properly, indicating the direct role of Hedgehog signaling in the development of the cochlea (Brown and Epstein, 2011). Using ganglion-specific *Shh−/−* mice, Bok et al. (2013) demonstrated that the auditory ganglion source of Shh regulates the timing of cell cycle exit and differentiation in mammalian cochlear HCs, and our previous study showed that Shh promotes the proliferation and HC formation of mouse embryonic inner ear progenitor cells (Zhao et al., 2006).

The generation of new HCs from the adjacent supporting cells after HC loss in the organ of Corti is a promising strategy for restoring hearing in mammals. In our previous study, we identified newly proliferated HCs, as indicated by positive staining for Myo7a and BrdU, by supplying recombinant Shh protein in the culture medium of neonatal mouse cochleae (Lu et al., 2013). We further demonstrated that the increased phosphorylation of pRb protein might be the reason for the mitotic HC regeneration in response to the addition of Shh protein to the culture medium and that the mitotically regenerated HCs could be either from the trans-differentiation of proliferated progenitor cells or from the HCs dividing along with the induction of progenitor genes, as was reported in 2005 (Mantela et al., 2005; Sage et al., 2005). To further investigate the effects of Hedgehog signaling on the supporting cells, we took advantage of conditional transgenic mice to up-regulate Hedgehog signaling specifically in Sox2+ supporting cells from P2 mice. Our current data show that forced Hedgehog signaling activation in supporting cells significantly increases the proliferation of Sox2+ supporting cells in the whole length of the cochlea after HC loss induced by neomycin exposure (Figure 4), and we showed that most of the regenerated HCs arose from the differentiation of proliferated supporting cells, as indicated by the increased number of EdU+Myo7a+ cells and the ratio of EdU+Myo7a+ cells to EdU+Sox2+ cells (Figure 4). We found that the mitotically regenerated HCs were mainly located in the pillar cell region, which is consistent with our previous results (Li et al., 2015), suggesting that the proliferated SCs and new HCs both originate from the Lgr5+ progenitor cells.

Wnt-responsive Lgr5+ supporting cells are HC progenitors in the mouse inner ear (Chai et al., 2012; Shi et al., 2012; Li et al., 2016; Waqas et al., 2016a,b; Cheng et al., 2017; Zhang et al., 2017). In the cochleae of neonatal mice, Lgr5 is expressed in a subset of supporting cells, including third-row Deiters’ cells, inner pillar cells, and medial inner phalangeal cells (Chai et al., 2011, 2012). In the damaged inner ear, Lgr5+ cells can regenerate HCs via proliferation and direct transdifferentiation (Wang et al., 2015; Zhang et al., 2017). In our current results, we report for the first time the effect of Hedgehog signaling on the postnatal Lgr5+ progenitor cells. Hedgehog signaling can promote the proliferation of postnatal Lgr5+ progenitor cells as indicated by the increase in the number and the diameter of spheres in the Shh-treatment group. Moreover, Hedgehog signaling is very important for the differentiation of Lgr5+ progenitor cells into HCs because inhibition of Hedgehog signaling with the Hedgehog antagonists significantly inhibited the formation of HCs (Figure 2). Interestingly, we observed that Myo7a+ HCs only appeared on the surface of the Lgr5+ spheres in both the control and Shh-treated groups, and the cells inside the spheres could not differentiate into HCs.

Our data showed that Hedgehog signaling fails to initiate cell proliferation or new HC formation in the intact cochlear sensory epithelium (Figure 3), but it does promote supporting cell proliferation and HC regeneration after neomycin-induced HC injury (Figure 4). This phenomenon might be related to changes in the gene expression profile in the progenitor cells after neomycin-induced HC loss. Compared with untreated Lgr5+ cells, neomycin-treated Lgr5+ cells had significantly greater HC regeneration capacity, which was accompanied by the down-regulation of Notch signaling, the up-regulation of Wnt signaling, and the down-regulation of HC formation inhibitors, including *Id1*, *Id2* and *Id3* (Zhang et al., 2017). As we known that the sorted Lgr5+ cells could proliferate and transdifferentiate into HCs *in vitro*, while *in situ* Lgr5+ progenitors could not automatically re-enter cell cycle *in vivo*, which strongly suggested that the sorted-Lgr5+ cells earned the proliferative potential might due to the loss of inhibition effects from the surrounding signaling. In our current experiments, the HC loss caused by neomycin treatment would definitely change the interaction between the HC and surrounding supporting cells, and resulted in the change of the local environment as well as the related signaling, which might serve as one promotion factor for the supporting cell proliferation and transdifferentiation. Thus, combined with the activation of Hedgehog we could observe obviously increased progenitor cell proliferation and HC formation showed in our manuscript.

In order to further understand the mechanism behind the mitotic HC regeneration induced by Hedgehog signaling in the cultured cochlear explants, we analyzed the gene expression profiles between the control samples and the Smo-OE samples. Among the differentially expressed genes between the control and Smo-OE groups, we found that the *Gli1* and *Hhip* genes, two downstream targets of Hedgehog signaling, were significantly up-regulated in the Smo-OE group, further confirming the constitutive activation of Hedgehog signaling in cochlear supporting cells in Smo-OE mice.

It has been reported that the Fgf, Wnt and Notch signaling pathways are involved in regulating proliferation, regeneration, and cell fate determination during inner ear development as well as the repair processes after HC loss in the cochlear sensory epithelium. In the current work, we observed differences in expression of multiple genes related to Fgf, Wnt, and Notch signaling between the control and Smo-OE group. Overall, compared with controls, 6 genes were up-regulated and 16 genes down-regulated in the Smo-OE group. Among these genes, *Wif1*, *Sfrp4* and *Dixdc1* have been reported to be related to Wnt signaling. *Wif1* encodes a protein that has been shown to inhibit Wnt signaling (Malinauskas et al., 2011) and to serve as an extracellular signaling molecule that plays a role during Xenopus embryonic nervous system development (Hsieh et al., 1999) and during mouse retinal development (Hunter et al., 2004). In the mammalian cochlea, the Wif1 protein regulates the development of unidirectional stereociliary bundle orientation (Dabdoub et al., 2003). Our data show that the expression of *Wif1* was down-regulated in Smo-OE mice and might serve as a proliferation-inhibiting gene. *Sfrp4*, which encodes a soluble modulator of Wnt signaling, was also down-regulated in the Hedgehog-activation group. *Dixdc1*, a gene that encodes a transcription factor that is a positive regulator of the Wnt signaling pathway (Chen et al., 2017), was up-regulated in the Hedgehog-activation group, and recent studies have shown that inhibition of Dixdc1 protein can suppress the proliferation of cancer cells (Chen et al., 2017; Zhong et al., 2017). Furthermore, *Mfng* and *Nhlh1* have been reported to be genes related to Notch signaling. *Mfng* encodes Manic Fringe, a negative regulator of Notch signaling (Veeraraghavalu et al., 2004; Golson et al., 2009), and this gene was up-regulated in the Hedgehog-activation group. A recent study has reported that the *Mfng* gene plays an important role in setting the boundary of the organ of Corti and in establishing sensory cell fate by modulating Notch signaling (Basch et al., 2016). Our results suggest that the *Mfng* gene might be a downstream target of the Hedgehog signaling pathway and might act as a mediator between the Hedgehog and Notch signaling pathways in the cochlear sensory epithelium.

*Nhlh1* encodes a helix-loop-helix protein that is indispensable for normal neuronal development by regulating the differentiation and cell fate decision of neural progenitor cells (Cogliati et al., 2002; Krüger and Braun, 2002; Krüger et al., 2004; Jahan et al., 2010). *Nhlh1* is also a downstream gene of Notch signaling (Ratié et al., 2014) and is assumed to be a gene that promotes HC formation (Żak et al., 2016). In our present study, we found that *Nhlh1* was up-regulated in the Smo-OE group, suggesting that Hedgehog-activation might promote HC differentiation via *Nhlh1* and Notch signaling. *Foxd3* is a tumor suppressor gene (Schmid and Müller, 2013) and has been reported to play roles in the pluripotency, proliferation, and differentiation of neural progenitor cells (Teng et al., 2008; Drerup et al., 2009). *Foxd3* was down-regulated in the Smo-OE group, suggesting that it might serve as a proliferation-inhibiting gene. The up-regulation of the cell cycle gene *Mki67*, which encodes the nuclear protein ki67 (Lin et al., 2016), in the Hedgehog-activation group further confirmed the phenomenon of enhanced proliferation of supporting cells observed in the immunofluorescence staining of Smo-OE cochlear explants (Figure 4).

Previous studies report that some genes, including *Atoh1*, *N-myc*, *L-Myc* and *Foxg1* are essential for HC formation during mammalian inner ear development (Zheng and Gao, 2000; Pauley et al., 2006; Kopecky et al., 2012). However, in our study, the RNA levels of these genes showed no significant difference between the control and Smo-OE group, suggesting that the effects of Hedgehog signaling on the proliferation of cochlear supporting cells and HC regeneration were not mediated by these genes.

In conclusion, in this work we show the effects of Hedgehog signaling on the Lgr5+ progenitor cells in postnatal cochlear explants and provide evidence for the mechanism underlying the proliferation and regeneration of these cells. The complicated interactions between the Fgf, Wnt, Notch and Hedgehog signaling pathways further highlight the possibility of promoting HC regeneration by manipulating multiple pathways at the same time, and our results suggest a number of genes that might serve as targets for promoting the proliferation and regeneration of HCs in the cochlear sensory epithelium, and more strategies should be designed for promoting the HCs regeneration and restoring hearing in the future.

## Author Contributions

HL, RC and WL conceived and designed the experiments. YC, XL, WN, YZ, LW, LZ, SZ, MT and SS performed the experiments. YC, XL, LG, HL, RC and WL analyzed the data. YC, RC, WL and HL wrote the article.

## Conflict of Interest Statement

The authors declare that the research was conducted in the absence of any commercial or financial relationships that could be construed as a potential conflict of interest.

## Footnotes

^1^http://david.abcc.ncifcrf.gov/home.jsp
