# Supplementary material for: Hedgehog Signaling Promotes the Proliferation and Subsequent Hair Cell Formation of Progenitor Cells in the Neonatal Mouse Cochlea
Source: Front Mol Neurosci. 2017 Dec 21;10:426. doi: 10.3389/fnmol.2017.00426 (PMC5742997; doi:10.3389/fnmol.2017.00426)
Supplement: Supplementary file 1 [file Table_1.pdf]

Table 1

PCR primer sequences used in these experiments

Gene	Forward sequence	Reverse sequence
Actb	5'-tctttgcagctccttcgttg-3'	5'-tccttctgacccattcccac-3'
Gli1	tctccgacccctccacag	agagatccttcagtgcagct
Gli2	actctcacctccatcagcac	ctcagcctcagtcttgacct
Gli3	ttttccctgccttccatcct	attacggtgtggggagatcc
Hhip	tcccgagaaagcaagtcaga	gcccactcatgacctcctg
Ptch1	atggccgcattgatccctat	tcttctgtcctcacgtctgt
Wif1	tacgagttcctgtctctgcg	ctgctaccccgtcttgtttg
Sfrp4	gtctatgaccgtggagtttgt	ttgctcaggtatgttgccag
Mfng	agtcatcctgctctgtcctg	gcccacatagacatcacggt
Nhlh1	ctctacgctgtccctctcac	tccccacccctccctttag
Dixdc1	ctccatcatcccgagactgg	ggtcgctcctgttcatcct
Mki67	caactactggaccctggacc	tccgaagctctgcatcatca
Spp1	gcttggcttatggactgagg	ccctttccgttgttgtcctg
Fgf11	actttgccagaaacagctcc	ttggtgacgatgcctttgag
Foxd3	acctggtcttagttccgttga	cttggttggcgtttctcctc
